# Supplementary material for: Controlling the synchronization and symmetry breaking of coupled bacterial pili on active biofilm carpets
Source: eLife. 2026 Jun 25;14:RP107609. doi: 10.7554/eLife.107609 (PMC13299615; doi:10.7554/eLife.107609)
Supplement: Peer review history

# Editor report: eLife Assessment

**DOI:** 10.7554/eLife.107609.3.sa0

**Authors:** Shashi Thutupalli

**Affiliations:** Simons Centre for the Study of Living Machines, National Centre for Biological Sciences (TIFR) India

**Keywords:** Important, Solid

This **important** study concerns the propagation of waves in bacterial biofilms, bridging active matter physics and bacterial biophysics. The experimental observations are **solid**, and the theoretical interpretation and model validation have been refined with revisions. This work will be of interest to microbiologists, biophysicists, and researchers studying collective behavior in biological systems.

# Referee report: Reviewer #1 (Public review)

**DOI:** 10.7554/eLife.107609.3.sa1

Summary:

Overall, this is an interesting paper. The authors identify several experimental knobs that can perturb mechanical wave behavior driven by pili feedback. They frame these effects in terms of nonreciprocal interactions. While nonreciprocity could indeed play a role, it raises the question of whether mechanical feedback might also contribute. Phenomenological models can be useful, but the model currently lack direct mechanistic insight. It would be more compelling to formulate the model around potential mechanochemical feedback, which could help clarify the underlying microscopic mechanisms.

Strengths:

Report of mechanical waves in bacterial collectives, mechanism has potential application in multicellular context such as morphogenesis.

Weaknesses:

A minor concern about the language of 'left-right asymmetry.' I believe the correct term is simply 'radial asymmetry' which is a distinct concept. Left-right is not well defined in the current context.

# Referee report: Reviewer #3 (Public review)

**DOI:** 10.7554/eLife.107609.3.sa2

Summary:

The revised manuscript presents a compelling study of radially propagating metachronal waves on the surface of Pseudomonas nitroreducens biofilms, combining experiments with two theoretical descriptions (a local phase-oscillator model and an active solid/active gel model). The central experimental findings-spiral/target/planar wave patterns, their controllability via water/PEG/temperature perturbations, and the correlation between frequency gradients and propagation direction-remain highly interesting and relevant to both bacterial biophysics and active-matter physics. The revised manuscript also adds substantial new material, including additional analyses of defect dynamics and clearer discussion of the relationship between the two models. The study continues to have a strong interdisciplinary appeal and the potential to stimulate further work on collective oscillations in biological active media.

Strengths:

The authors have substantially addressed the major conceptual issue raised in the previous round by clearly distinguishing between nonreciprocity and frequency gradients / global asymmetry. This clarification significantly improves the theoretical interpretation and resolves an important source of confusion in the original version.

The revised manuscript also improves the connection between the phase-oscillator and active-solid descriptions. In particular, the authors now explain more explicitly how the phase variable is defined in the reduced oscillatory dynamics of confined biofilm motion, and they state that they added a schematic illustration and simulation details (including parameter values and the elastic-force definition) to improve reproducibility. This directly addresses one of my previous major concerns.

A notable improvement is the newly added defect-based analysis of waveform transitions (spiral -> target -> planar). The revised text argues that defect motility is a key control parameter, linked experimentally to moisture-dependent elasticity and theoretically to nonreciprocity / defect-pair stability. This provides a more concrete mechanistic bridge between experimental perturbations and the modeling framework than in the previous version.

The manuscript now gives a clearer experimental-theoretical narrative for how environmental manipulations (drying, water addition, PEG, heating) affect wave patterns through changes in effective elasticity and activity, including a useful distinction between short-timescale and long-timescale temperature effects. This added discussion strengthens the biological interpretation and makes the modeling assumptions easier to follow.

Weaknesses:

The main remaining limitation is the level of quantitative correspondence between theory and experiment. The revised manuscript now provides a stronger qualitative/mechanistic link, but the mapping between model parameters (e.g., effective coupling terms / elasto-active parameters) and directly measurable biofilm properties is still limited. The authors acknowledge this point, and I agree that it is technically challenging in the present system. However, this means the theoretical framework is currently most convincing as an effective mechanistic model rather than a quantitatively predictive one.

Relatedly, some conclusions about parameter-level control (especially in connecting moisture/temperature manipulations to specific model parameters) remain qualitative. I do not view this as fatal, but I recommend that the manuscript clearly state this scope and avoid overstating the quantitative predictive power of the theory.

Although the terminology has improved compared with the original version, the revised manuscript still uses "left-right asymmetry" in places where the underlying geometry and symmetry are more general (e.g., radial inward propagation in circular colonies). Since this wording was one of the original points of confusion, I suggest one final pass to ensure the symmetry language is consistently precise throughout the main text and figure captions.

# Author comment: Author response

**DOI:** 10.7554/eLife.107609.3.sa3

**Authors:** Baha Altın, Enes Talha Günay, İlker Yusuf Yaman, Alp Ünlü, Yiğithan Gediz, Neslihan Gedik, Bora Karataş, Mustafa Başaran, Coşkun Kocabaş, Şahin Kaya Özdemir, Askin Kocabas

**Affiliations:** Koç University Istanbul Turkey; Koç University Istanbul Turkey; Koç University Istanbul Turkey; Koç University Istanbul Turkey; Koç University Istanbul Turkey; Boğaziçi University İstanbul Turkey; Koç University Istanbul Turkey; Koç University Istanbul Turkey; University of Manchester Manchester United Kingdom; Saint Louis University Saint Louis United States; Koç University Istanbul Turkey

The following is the authors’ response to the original reviews.

**eLife Assessment**

This important study concerns the propagation of waves in bacterial biofilms, bridging active matter physics and bacterial biophysics. While the experimental observations are solid, the theoretical interpretation and model validation are currently incomplete and require further refinement. This work will be of interest to microbiologists, biophysicists, and researchers studying collective behavior in biological systems.

In the revised manuscript, we have added new experimental results that strengthen the connection between our observations and the modeling framework used to interpret the collective oscillations. We have not introduced a new theoretical model; rather, we employed established active matter models and sought to link the observed phenomena to these frameworks. In particular, our new data demonstrate that the transition between the motile and biofilm-forming states specifically modulates the elasticity and elasto active coupling of the bacterial structure. This behavior is in excellent agreement with the predictions of the active solid model. All the experimental details are given below. We believe that the revised version of the manuscript now establishes this connection more clearly and convincingly.

**Public Reviews:**

**Reviewer #1 (Public review):**

Summary:

Overall, this is an interesting paper. The authors have found multiple experimental knobs to perturb a mechanical wave behavior driven by pilli feedback. The authors framed this as nonreciprocal interactions - while I can see how nonreciprocity could play a role - what about mechanical feedback? Phenomenological models are fine, but a lack of mechanistic understanding is a weakness. I think it will be more interesting to frame the model based on potential mechanochemical feedback to understand microscopic mechanisms. Regardless, more can be done to better constrain the model through finding knobs to explain experimental observations (in Figures 3, 4, 5, and 7).

We thank the reviewer for the positive assessment and for highlighting this important point. The reviewer is correct that the phenomenological Kuramoto-based model does not explicitly show the detailed cell–cell interactions. However, the active solid model is formulated on detailed elastic couplings and active forces, which inherently represent mechanical feedback within the biofilm structure. In this framework, nonreciprocity emerges naturally from the tensorial nature of active forces between bacteria—a concept already well established in the active matter literature. Importantly, this mechanism is purely mechanical and closely parallels nonreciprocal hydrodynamic interactions among active particles, which also arise from tensorial couplings.

In our system, elastic interactions within the biofilm matrix, combined with pilus-generated active forces, provide a natural origin for nonreciprocal interactions. To further validate this, we improved our imaging to record single-cell dynamics both at the colony edge and on the biofilm surface. (new supplementary Video). These experiments show that motile bacteria at the leading edge of the biofilm structure do not generate waves, whereas stationary bacteria within the biofilm display local oscillations within the elastic network. This observation supports the view that collective oscillations are a property of the elastic biofilm state rather than of freely motile cells.

Moreover, the main control parameter for these oscillations is the ratio between elastic strength and the active force generated by pili. In the active solid model, this ratio is captured by the parameter π and alpha terms. Experimentally, we can tune this ratio simply by adding or removing water from the biofilm, thereby modulating its elasto active coupling. We further motivated the controllability of this feature experimentally. We let the plate dry nonuniformly and observed that the transition between spiral target and plane waves could emerge spontaneously across the plate (see Figure 3a). This observation also states the importance of moisture in the biofilm. Starting from this point we established the connection between experimental observation and modelling. In our new simulations we also noticed that the transition from spiral to target wave is particularly driven by merging processes of different topological charges +/- 1 spiral pairs. This critical point was also confirmed by modelling which links the process to elasto active coupling. Further we supported our claim by imagining the edge and the biofilm structure. These new results clarify that elastic structure of the biofilm is critically important (Supplementary Figure 3). We have clarified this mechanistic link in the revised manuscript and rewritten the relevant sections to make this connection explicit.

Modification in the manuscript:

“To gain deeper insight into the mechanisms underlying wave formation, we imaged the dynamics of individual bacteria from the fingering regions toward the center of the biofilm. This distinction is critical because, unlike the biofilm center, the edges do not generate waves. We observed that bacteria near the fingering regions remain motile and exhibit collective flow. In contrast, bacteria at the biofilm center are surface-attached and undergo periodic lifting motions. This behavior strongly resembles Mexican-wave dynamics.”

“We further found that the central region of the biofilm is mechanically more elastic, whereas the edge regions—where wave formation is absent—are motile. These observations suggest that gradual biofilm maturation is a key factor that transforms motile bacteria into a periodically moving but spatially constrained state. Consistent with this picture, the PAO1 strain, which has a strong biofilm-forming capability, completely suppresses surface oscillations. In contrast, the PA14 strain exhibits intermediate behavior, sustaining a partial transition between motile and locally constrained dynamics. Remarkably, signatures of this transition and wave generation are already detectable at the earliest stages of finger formation.”

Strengths:

The report of mechanical waves in bacterial collectives. The mechanism has potential application in a multicellular context, such as morphogenesis.

We thank the reviewer for the positive assessment and for highlighting this potential broad impact of our findings.

Weaknesses:

My most serious concern is about left-right symmetry breaking. I fail to see how the data in Figure 6 shows LR symmetry breaking. All they show is in-out directionality, which is a boundary condition. LR SM means breaking of mirror symmetry - the pattern cannot be superimposed on its mirror image using only rigid body transformations (translation and rotation) - as far as I am aware, this condition is not satisfied in this pattern-forming system.

We thank the reviewer for pointing out this critical issue. We acknowledge that we overlooked the distinction between biological and physical definitions of left–right symmetry in our initial submission, and we agree that our terminology was confusing.

In developmental biology, the term “left–right symmetry breaking” is often used to describe asymmetric flows generated by nodal cilia, which subsequently establish developmental asymmetry. This usage differs fundamentally from the physical definition of mirror symmetry breaking, which refers to chirality switching upon mirror reflection. As the reviewer correctly noted, our system does not exhibit mirror symmetry breaking in this strict physical sense.

To avoid confusion, we have revised the manuscript and replaced the term left–right symmetry breaking with left–right asymmetry between the edge and the center of the biofilm. This asymmetry arises from frequency gradients across the biofilm and is not a trivial boundary effect. For circular colonies, this phenomenon is more accurately described as radial asymmetry. We have rewritten the relevant sections of the manuscript to clarify this distinction and prevent misinterpretation.

**Reviewer #2 (Public review):**

Summary:

This manuscript by Altin et al. examines the dynamics of bacterial assemblies, building on previously published work documenting mechanical spiral waves. The authors show that the emergent dynamics can be influenced by various factors, including the strain of bacteria and water content in the sample. While the topic of this paper would be of broad interest, and the preliminary results are certainly interesting, various aspects of this paper are underdeveloped and require further exploration.

Strengths:

One of the nice features of this system is the ability to transition between the different states based on the addition or withdrawal of water. The authors use a similar experimental model system and mathematical model to previously published work (Reference 49), but extend by showing that the behaviour can be modified through simple interventions. Specifically, the authors show that adding water droplets or drying the sample through heating can result in changes in the observed wave structure. This represents a possible way of controlling active matter.

The mathematical model proposed in this paper involves a phase-oscillator model of Kuramotostyle coupling (similar to previously reported models). A non-reciprocal phase lag is introduced in order to facilitate the patterns seen in experiments. The qualitative agreement in the behaviour is quite striking, showing both spiral waves and travelling waves.

We thank the reviewer for the positive assessment and for pointing out areas that required further development. The reviewer is correct that our work builds on previously reported bacterial spiral wave systems; however, there are several significant differences that we now emphasize more clearly in the revised manuscript.

First, our study involves a different bacterial species and reveals a distinct dynamical process: the waves we report are strictly localized on the surface of the biofilm, in contrast to the bulk oscillations detected through density fluctuations in the earlier work (Ref. 49). The surface waves in our system resemble “Mexican wave”-like motions, in which surface bacteria periodically lift upward. To highlight this key distinction, we performed new imaging experiments that directly visualize this process. (New Video 5 and 6, Author response image 1).

Second, we systematically compared different bacterial strains, including pathogenic species such as *P. aeruginosa* PA14 and PAO1, alongside our BSL-1 strain. This comparative approach demonstrates that the observed phenomenon spans strains with different pathogenicity levels, and genetic variations while also showing that our strain provides a safer and more broadly usable model system for laboratory investigations.

Third, the modeling frameworks differ. Whereas the referred study relied primarily on phase models similar to those used in cilia systems, we combine a delayed Kuramoto-style oscillator model with an active solid model. This combination provides both a phenomenological description and a physical interpretation of the collective dynamics. We acknowledge that, in the original submission, the physical interpretation of the model in relation to our experimental system was underdeveloped. In the revision, we have now established this link explicitly through the elasticity and elasto active coupling of the biofilm. Specifically, we show that the transition from motile to biofilm states is accompanied by changes in elasticity, which directly influence the observed transitions between different types of wave defects. This connection is consistent with prior theoretical works and has even been only studied in robotic active matter systems.

Together, these clarifications and new results reinforce the novelty of our findings and establish a stronger connection between the experiments and the modeling framework.

**Author response image 1.** Comparison between the elastic biofilm core and the motile colony edge. Highresolution video recordings revealing individual bacterial motion highlight the key physical differences driving wave-generating. Time-lapse snapshots show that bacteria at the colony edge move freely and form fingering structures, whereas bacteria in the elastic central biofilm periodically lift vertically, producing a Mexican-wave–like collective motion across the surface. See new Video

Weaknesses:

The principal observation of the paper - that spiral waves emerge in these systems and can be controlled in various ways - is not linked to microscale dynamics at the cell level. It is recognised that hydrodynamics can introduce non-reciprocity, an essential ingredient of this model. However, in this work the authors have not identified a physical mechanism for the lag, e.g., either through steric interactions or hydrodynamic disturbances. This is also relevant in the phase oscillator modelling section. In low Reynolds number flows, dynamics are instantaneously determined. In this light, what does the phase lag term represent?

The reviewer is correct that, at low Reynolds numbers, fluid dynamics are instantaneous and do not generate real temporal delays. However, nonreciprocity in hydrodynamic interactions can still emerge from the tensorial structure of the Blake–Oseen Green’s function. In this formalism, the effective asymmetry can be represented mathematically as a phase-lag–like term. This has been theoretically demonstrated in Ref.40. While this is not a literal time delay, it functions analogously by breaking odd symmetry in the coupling.

In our system, strong long-range hydrodynamic interactions are absent, as the bacteria are embedded in an elastic biofilm matrix. Instead, the dominant interactions are active elastic couplings mediated by pili and biofilm structure. The elastic solid model behaves in a way that is conceptually similar to the hydrodynamic case: pili-induced deformations of the elastic medium produce anisotropic stresses that play a role analogous to the tensorial hydrodynamic Green’s function. Thus, the phase-lag term in our Kuramoto-based model can be interpreted as an effective representation of these nonreciprocal elastic interactions.

We have clarified this point in the revised manuscript by explicitly connecting the phenomenological phase-lag term to the underlying elastic coupling in biofilms.

What is the origin of the coupling term, b? Can this be varied systematically or derived from experimental measurements or parameters?

The term *b* represents the enhanced elasto-active coupling of the pili process. The length of the Pili varies, and the elongated Pili has more potential to modulate the coupling between bacteria which is known to depend on a critical threshold. This process resembles the pinning dynamics and is driven by the activity of molecular motors within the pili machinery. However, the detailed mechanisms that set the effective coupling strength remain highly complex and are not yet fully understood.

At present, we do not have a direct way to systematically manipulate *b* in experiments. A major technical limitation is the nanoscale nature of type IV pili: these protein assemblies are extremely small and difficult to monitor or manipulate directly. Even basic tools such as GFP-based labeling have proven challenging to implement, which restricts our ability to track the detailed dynamics of these structures in live biofilms.

While we cannot currently derive *b* directly from experimental parameters, we emphasize in the revised manuscript that *b* should be understood as an effective parameter capturing the excitability of pili retractions. We also highlight this limitation and note that future advances in molecular imaging and manipulation of pili will be essential for quantitatively linking *b* to microscopic processes.

Classification of wave properties is an important aspect of this paper, but is not accomplished in a quantitative sense. What is the method for distinguishing between travelling and spiral waves? There is a range of quantitative tools that could be used to investigate these dynamics (and also compare quantitatively with the models). For example, examining the correlation functions and order parameters could assist with the extraction of wave features (see extensive literature on oscillator models).

We thank the reviewer for emphasizing this important point. In the revised manuscript, we have incorporated the classic Kuramoto order parameter (S) to characterize the dynamics in our model simulations. However, this metric is not directly applicable to our experimental system, because we cannot resolve the phase of individual bacteria at large scales.

Instead, we have focused on a flux-based parameter, as previously used in Ref. 40, which can be measured experimentally from collective surface dynamics. Interestingly, we find that the directional flux extracted from our experimental movies closely matches the trends predicted by the model order parameter. We suspect that this similarity arises from the combination of our optical illumination method and the characteristic surface modulations of the biofilm. While we currently lack a rigorous theoretical justification for this correspondence, so we want to keep this discussion in the review document.

In summary, we now use the classic Kuramoto order parameter in simulations and rely on the experimentally accessible flux measure for our experimental data. This dual approach allows us to compare model and experiment in a consistent manner.

**Author response image 2.** Critical order parameters of the coupled biofilm system. (a) The Kuramoto global order parameter increases continuously as the system becomes globally synchronized. In contrast, in the nonreciprocally coupled system the order parameter saturates at a critical level. (b) In the experimentally observed biofilm, however the flux generated by the coupled oscillations provides a more appropriate measure of synchronization. Blue curves indicate directionally propagating planar waves, red curves correspond to spiral wave formation, and green curves represent the globally synchronized reciprocal system.

**Author response image 3.** Comparison of flux profiles of the simulations with experimental measurements. Directional optical illumination enhances the flux term on the surface of the biofilm.

The methodology of changing the dynamics through moisture content appears to be slightly underdeveloped, e.g., adding water involves a droplet, and removing water is accomplished by heating (which presumably could cause other effects). Could the dynamics not be controlled more directly by varying the humidity?

We thank the reviewer for this valuable suggestion. Our results indicate that water content in the biofilm plays a key role in driving the transition to the biofilm state by modulating its elasticity. During the initial submission, we did not know how to systematically vary humidity without simultaneously altering temperature. Standard approaches typically involve water evaporation in controlled chambers, which inherently changes both parameters.

Following the reviewer’s recommendation, we first measured the ambient moisture levels inside closed culture plates. To our surprise, the relative humidity was already ~98%, leaving virtually no room to increase it further. We then attempted to decrease humidity by flowing dry synthetic air, but even under these conditions we could not reduce it below ~85%, and achieving this required unrealistically high flow rates. Moreover, we noticed that in closed-lid NGM plates, evaporation is already substantial, and when the lid is left open the evaporation rate reaches ~1 µm/s. This rapid surface thinning severely limits the quality of long-term time-lapse imaging.

Taken together, these technical constraints explain why we have to reliy on localized perturbations such as water droplets and heating rather than global humidity control. We have clarified this point in the revised manuscript and now explicitly discuss both the challenges and limitations of humidity-based approaches.

At the same time, the authors also mention that temperature itself plays a role in shaping the behaviour. What is the mechanism for this? Is it just through evaporation? Since the frequency increases with temperature, could it just be that activity increases with temperature?

We thank the reviewer for raising this critical point. We believe that temperature has two distinct impacts operating on different timescales.

Short timescale (~minutes): We observed that biofilm oscillations respond to temperature changes very rapidly and in a reversible manner. This timescale is too short to be explained by modulation of water content or bulk elasticity of the biofilm. Instead, we attribute the immediate frequency increase to enhanced biological activity of the bacteria at elevated temperatures.

Long timescale (~tens of minutes to hours): During processes such as the transition from planar to spiral waves, prolonged heating can significantly alter the biofilm structure. These changes are not reversible and likely involve modifications of elasticity and other structural properties.

In the modeling framework, the short-timescale effect is represented as an increase in the active force term, while the long-timescale effect is captured by concurrent changes in both the active force and the elastic properties of the biofilm. We have clarified this mechanism and its representation in the revised manuscript.

**Reviewer #3 (Public review):**

Summary:

This manuscript presents a novel investigation into unidirectionally propagating waves observed on the surface of Pseudomonas nitroreducens bacterial biofilms. The authors explore how these waves, initially spiral in form, transition into combinations of spiral, target, and planar patterns. The study identifies the periodic extension-retraction cycles of type IV pili as the driving mechanism for wave propagation, which preferentially moves from the colony's edge to its center. Furthermore, the manuscript proposes two theoretical models-a phase-oscillator model and a continuum active solid model-to reproduce these phenomena, and demonstrates how external manipulations (e.g., water droplets, temperature, PEG) can control wave patterns and direction, often correlating with oscillation frequency gradients. The work aims to bridge the fields of activematter physics and bacterial biophysics by providing both experimental observations and theoretical frameworks for understanding these complex biological wave phenomena.

We thank the reviewer for the positive assessment of our work and for highlighting both the novelty and the key contributions of our study.

Strengths:

The experimental discovery of unidirectionally propagating waves on bacterial biofilms is highly intriguing and represents a significant contribution to both microbiology and active-matter physics.

The detailed observations of wave pattern transitions (spiral to target to planar) and their response to various environmental perturbations (water, temperature, PEG) provide valuable empirical data. The identification of type IV pili as the driving force offers a concrete biological mechanism. The observed correlation between frequency gradients and wave direction is a compelling finding with potential for broader implications in understanding biological pattern formation. This work has the potential to stimulate further research in the collective behavior of living systems and the physical principles underlying biological organization.

We thank the reviewer once again for emphasizing the importance of wave directionality. We also believe that this phenomenon may provide insight into early symmetry-breaking processes observed in developmental biology, where oxygen or nutrient gradients in dense environments could play a similar role.

Weaknesses:

The manuscript attempts to link unidirectional wave propagation to non-reciprocal couplings but ultimately shows that the wave direction is determined by the gradient of the oscillation frequency. The couplings in the two theoretical models are both isotropic and thus cannot dictate the wave direction. A clear distinction should be made between non-reciprocity as a source of wave generation and non-uniformity as a controlling factor of wave direction.

We greatly appreciate the reviewer’s careful evaluation, particularly for highlighting this important and often confusing distinction. The relationship between nonreciprocity, spontaneous symmetry breaking, and frequency gradients has also been a challenging concept for us and required significant effort to clarify.

Recent theoretical studies have established that traveling wave formation requires nonreciprocity, which provides a framework for understanding phenomena ranging from spiral to target and planar waves. In our system, nonreciprocity arises between the displacement field (*U*) and the pili force vector (*P*): as a result in broken phase *U* effectively “chases” *P*, breaking PT symmetry locally and thereby enabling the generation of local directional flux and traveling waves. In this sense, nonreciprocity is essential for travelling wave generation and spontaneous symmetry breaking in either direction.

However, we now agree that global directionality (always from right to left, or edge to center) is set by an independent factor—namely, the oscillation frequency gradient across the biofilm. Thus, while nonreciprocity determines whether waves can travel, frequency gradients determine the large-scale direction in which they propagate. Put differently, PT symmetry is already broken spiral waves due to nonreciprocity, but global asymmetry (frequency gradients) is required to align the overall propagation in one direction.

We have clarified this distinction in the revised manuscript, emphasizing that nonreciprocity is a necessary ingredient for travelling wave generation, whereas global asymmetry controls global wave direction.

Modification in the manuscript:

“We should note that traveling waves indicate broken PT symmetry between these fields triggered by nonreciprocity, with spiral waves serving as a classic signature of this phenomenon. A further transition from spiral to planar waves reflects an overall asymmetry in the frequency profile, which is not directly related to PT-symmetry breaking.”

The relationship between the phase oscillator model and the active solid model is unclear. Given that U and P are both dynamical variables evolving in three-dimensional space, defining the phase Φ precisely in the phase space spanned by U and P could be challenging. A graphical illustration of the definition of Φ would be beneficial. To ensure reproducibility of the numerical results, the parameter values used in the numerical simulations and an explicit definition of the elastic force in the active solid model should be provided.

We agree with the reviewer that the relationship between the phase oscillator model and the active solid model can be confusing, but establishing this link is essential to connect different modeling approaches in the literature. As the reviewer notes, in a fully three-dimensional setting with freely moving bacteria, defining the oscillation phase (Φ) in the phase space spanned by *U* and *P* is indeed complicated.

However, our recent imaging results show that bacteria within the biofilm do not undergo large translational motions but instead exhibit periodic “Mexican wave”-like oscillations. These oscillations are confined to a restricted phase space, which allows us to define Φ in a straightforward way. In this context, the phase oscillator model becomes a natural reduction of the dynamics.

Similarly, in the active solid (or active gel) model, we can plot not only the displacement and force vectors but also the local phase, which shows strong agreement with the phenomenological Kuramoto-style model. To make this connection clearer, we have now included a schematic illustration in the revised manuscript that explicitly shows how Φ is defined in the reduced phase space, and we provide the parameter values used in the simulations as well as the explicit definition of the elastic force in the active solid model to ensure reproducibility.

The link between the theoretical models and experimental results is weak. For example, the propagation of the kink from the lower to the higher part of the surface (Figure 1e) could be addressed within the framework of the active solid model. The mechanism of transition from spiral to target waves (Figure 3a, b) requires clarification, identifying which model parameter is crucial for inducing this transition. The wave propagation toward the lower frequency side is numerically demonstrated using the phase oscillator model, but a physical or intuitive explanation for this phenomenon is missing. Also, the wave transitions induced by the addition of water droplets and temperature rise are not linked to specific parameters in the theoretical models.

We thank the reviewer for highlighting this important weakness, which was also consistently noted by the other reviewers. We fully agree that the link between our theoretical models and experimental results required significant strengthening.

With improved imaging in the revised study, we were able to uncover additional connections that help establish this link more clearly. We acknowledge that our ability to measure detailed biofilm parameters is limited, which restricts us from providing fully quantitative mappings. Nonetheless, based on the reviewers’ suggestions, we carried out additional imaging and simulations to compare bacterial dynamics at the colony edge and within the biofilm surface. These data confirm that cells within the biofilm undergo restricted, “Mexican wave”-like oscillations, emphasizing the critical role of elasticity in governing the collective dynamics.

Experimentally, we found that adding water or PEG, or alternatively inducing drying, strongly modulates the effective elasticity of the biofilm. Within the active solid framework, elasticity and the elasto-active coupling are the key parameters controlling the system. By tuning these parameters in simulations, we could reproduce the qualitative transitions observed experimentally. Specifically, we observed that:

At low elasticity, topological defects are mobile and can move, merge, or annihilate, leading to the emergence of planar waves.

At high elasticity, defects remain pinned, across the biofilm surface, dominating the dynamics.

These observations suggest that the motility of defects is the crucial parameter governing the transition between spiral, target, and planar waves. Although we cannot independently manipulate each parameter in experiments, varying the moisture content provides an effective and experimentally accessible control.

Finally, our simulations and new analyses reveal that spiral defect cores can move and merge to form target waves or annihilate entirely—processes that we also observe experimentally. This rich dynamical behavior underscores the importance of elasticity in shaping pattern transitions, and we believe it warrants further theoretical exploration. We have clarified this connection and its implications in the revised manuscript.

First, we compare defect dynamics in both Kuramoto-based simulations and the active solid model. Both systems exhibit similar defect-survival behavior. As shown in the review , pairs of unlike (+/−) defects can stably persist only at high nonreciprocity. We further quantify this behavior by plotting the separation distances between unlike defect pairs and find that short-range defect separations are possible exclusively in the high-nonreciprocity regime Supplementary Figure 11.

This high-nonreciprocity regime corresponds to the dry biofilm state. Increasing moisture reduces elasticity, leading to the loss of stable defect dynamics and promoting the annihilation of unlike defect pairs, which in turn drives the system toward target-wave formation and ultimately planar waves. Conversely, heating the biofilm removes water, enhances elasticity, and increases the system’s ability to sustain closely separated defect pairs.

Experimentally, we further observe that removing water by heating enhances surface nonuniformities, which readily trigger defect-pair formation. To investigate this mechanism, we performed additional simulations in which local nonuniformities were introduced Supplementary Figure 12. Consistent with experiments, defect-pair generation occurs only at high nonreciprocity, where pairs of unlike defects can be stably maintained. Experimental observation (Author response image 4) also show that surface nonuniformities on the biofilm surface similarly trigger the formation of closely separated defect pairs. We have updated the details of the defect dynamics in the revised manuscript to clarify the transition between these waves.

**Author response image 4.** Experimental observation showing that small surface nonuniformities on the biofilm surface trigger the formation of closely separated defect pairs. Arrows indicate the position of the nonuniformities

Modification in the manuscript:

Defect dynamics controlling the transition between spiral to target waves

“To better understand the dynamics of the transition between different form of the waves we focused on numerical simulations. We noticed that the motility of defects is the crucial parameter governing the transition between spiral, target, and planar waves varying the moisture content provides an effective and experimentally accessible control this motility. Our analyses revealed that spiral defect cores can move and merge to form target waves or annihilate entirely—processes that we also observe experimentally. This rich dynamical behavior underscores the importance of elasticity in shaping pattern transitions. First, we compare defect dynamics in both Kuramotobased simulations and the active solid model. Both systems exhibit similar defect-survival behavior. As shown in Supplementary Figure10, pairs of unlike (+/−) defects can stably persist only at high nonreciprocity. We further quantify this behavior by plotting the separation distances between unlike defect pairs and find that short-range defect separations are possible exclusively in the high-nonreciprocity regime (Supplementary Figure11). This high-nonreciprocity regime corresponds to the dry biofilm state. Increasing moisture reduces elasticity, leading to the loss of stable defect dynamics and promoting the annihilation of unlike defect pairs, which in turn drives the system toward target-wave formation and ultimately planar waves. Conversely, heating the biofilm removes water, enhances elasticity, and increases the system’s ability to sustain closely separated defect pairs. Experimentally, we further observe that removing water by heating enhances surface nonuniformities, which readily trigger defect-pair formation (Supplementary Video9). To investigate this mechanism, we performed additional simulations in which local nonuniformities were introduced (Supplementary Video12-13). Consistent with experiments, defect-pair generation occurs only at high nonreciprocity, where pairs of unlike defects can be stably maintained. Experimental observation (Supplementary Video9) also show that surface nonuniformities on the biofilm surface similarly trigger the formation of closely separated defect pairs.”

All the recommended points have been addressed in the revised manuscript.
